# Vitamins and functional nutrients for extremely low birth weight infants

**DOI:** 10.1186/1824-7288-41-S1-A38

**Published:** 2015-09-24

**Authors:** Gennaro Salvia, Sabino Moschella, Violante Fonterico, Sonia Di Lena, Giuseppina Mazzarella

**Affiliations:** 1 Neonatal Intensive Care Unit, Buon Consiglio Fatebenefratelli Hospital, Naples, Italy

## 

While the need for appropriate post natalgrowth provides many studies focused on macronutrients in the nutrition of preterm infants, the concern for the quality of development of micro premies increases the interest in micronutrients, vitamins and trace elements. Moreover, the ongoing challenge to avoid serious complications of extreme prematurity requires investigations on functional nutrients especially those with antioxidant effect or immunomodulatory properties.

Despite the lack of defined recommendations, much evidence is available.

The antioxidant properties of vitamin A and vitamin E make these elements important in the nutrition of ELBW infants who are born with a strong deficit of these vitamins. Low plasma vitamin A concentrations increases the risk of chronic lung disease and long-term respiratory disabilities in preterm infants. Vitamin E counteracts peroxidation of polyunsaturated fattyacids in lipid layers of cell membranes[1]. 

Preterm infants are exposed to a special risk for metabolic bone disease also due to maternal deficiency and difficulty achieving adequate enteral intake of calcium, phosphorus, and vitamin D. The link between vitamin D supplementation, serum 25-OH-D concentration and long term outcome inpreterminfants are poorly understood. Some findings supportthe opinion that a vitamin D intake of 800-1000 IU/day is necessary in preterm infants while higher doses are needed in newbornof mothers with vitamin D deficiency [2]. Furthermore, vitamin D plays a rolein physiological processes that affect neuromuscular and immune functions, heart, lung, pancreas, and brain.

Many trials reported enhanced visual acuity and better cognitive performance in the short-term assessments of preterm infants fed formulas supplemented with long chain polyunsaturated fattyacids(LCPUFA) [3-5]. However, there are many questions about the appropriate dosages and some doubts on the long-term advantages [6,7]. Among strategies for the prevention of NEC and sepsis through the use of immunonutrients, lactoferrin[8] and probiotics[9] are getting a place in clinical practice. Other studies will be needed to assess the extension of the evidence currently observed, especially for neonatesin low NEC rates settings. To date are available only weak evidence about the efficacy of arginine and glutamineto prevent intestinal mucosal injury in high risk infants [10]. Lutein supplementation for preterm infants raises plasma concentrations to those observed in human milk fed term infants and ensures the benefit of anti oxidant effect of carotenoidson immature retina and developing brain. Larger trials are needed to determine the best methods of administration to affect the retinopathy of prematurity[11].
